# Comparison of Interpolation Methods in the Diagnosis of Carpal Tunnel Syndrome

**DOI:** 10.4274/balkanmedj.2017.1314

**Published:** 2018-09-21

**Authors:** Veysel Alcan, Murat Zinnuroğlu, Gülçin Kaymak Karataş, Elliot Bodofsky

**Affiliations:** 1 Dikmen Vocational and Technical Anatolian High School, Ankara, Turkey; 2 Department of Physical Medicine and Rehabilitation, Gazi University School of Medicine, Ankara, Turkey; 3 Department of Physical Medicine and Rehabilitation, Cooper Medical School of Rowan University, Camden, New Jersey, USA

**Keywords:** Carpal tunnel syndrome, diagnosis, interpolation, neural conduction

## Abstract

**Background:**

Diagnosis of carpal tunnel syndrome is based on clinical symptoms, examination findings, and electrodiagnostic studies. For carpal tunnel syndrome, the most useful of these are nerve conduction studies. However, nerve conduction studie can result in ambiguous or false-negative results, particularly for mild carpal tunnel syndrome. Increasing the number of nerve conduction studie tests improves accuracy but also increases time, cost, and discomfort. To improve accuracy without additional testing, the terminal latency index and residual latency are additional calculations that can be performed using the minimum number of tests. Recently, the median sensory-ulnar motor latency difference was devised as another way to improve diagnostic accuracy for mild carpal tunnel syndrome.

**Aims:**

The median sensory-ulnar motor latency difference, terminal latency index, and residual latency were compared for diagnostic accuracy according to severity of carpal tunnel syndrome.

**Study Design:**

Diagnostic accuracy study.

**Methods:**

A total of 657 subjects were retrospectively enrolled. The carpal tunnel syndrome group consisted of 546 subjects with carpal tunnel syndrome according to nerve conduction studie (all severities). The control group consisted of 121 subjects with no hand symptoms and normal nerve conduction studie. All statistical analyses were performed using SAS v9.4. Means were compared using one-way ANOVA with the Bonferroni adjustment. Sensitivity, specificity, positive predictive value, and negative predictive value were compared, including receiver operating characteristic curve analysis.

**Results:**

For mild carpal tunnel syndrome, the median sensory-ulnar motor latency difference showed higher specificity and positive predictive value rates (0.967 and 0.957, respectively) than terminal latency index (0.603 and 0.769, respectively) and residual latency (0.818 and 0.858, respectively). The area under the receiver operating characteristic was highest for the median sensory-ulnar motor latency difference (0.889), followed by the residual latency (0.829), and lastly the terminal latency index (0.762). Differences were statistically significant (median sensory-ulnar motor latency difference being the most accurate). For moderate carpal tunnel syndrome, sensitivity and specificity rates of residual latency (0.989 and 1.000) and terminal latency index (0.983 and 0.975) were higher than those for median sensory-ulnar motor latency difference (0.866 and 0.958). Differences in area under the receiver operating characteristic curve were not significantly significant, but median sensory-ulnar motor latency difference sensitivity was lower. For severe carpal tunnel syndrome, residual latency yielded 1.000 sensitivity, specificity, positive predictive value, negative predictive value and area beneath the receiver operating characteristic curve. Differences in area under the receiver operating characteristic curve were not significantly different.

**Conclusion:**

The median sensory-ulnar motor latency difference is the best calculated parameter for diagnosing mild carpal tunnel syndrome. It requires only a simple calculation and no additional testing. Residual latency and the terminal latency index are also useful in diagnosing mild to moderate carpal tunnel syndrome.

Carpal tunnel syndrome (CTS) is a complex syndrome caused by compression of the median nerve beneath the transverse carpal ligament (1). CTS is characterized by paresthesia, pain, atrophy, weakness, and sensory abnormalities in median nerve innervation (2). Early diagnosis of CTS increases the probability of successful treatment. Diagnosis of CTS is based on clinical symptoms, physical examination findings, and electrodiagnostic tests, primarily nerve conduction studies. Clinical tests can identify probable cases. Electrodiagnostic findings improve diagnosis (3). Electrodiagnostic tests are used to confirm the diagnosis of CTS and exclude other possible causes, including cervical radiculopathy or peripheral neuropathy (4).

However, several studies show that routine electrodiagnostic tests have limited sensitivity and specificity for mild CTS (4,5,6,7,8,9). An expensive, uncomfortable test with inaccurate results is not helpful. Therefore, additional calculations utilizing the minimum number of tests to improve accuracy are crucial. These tests include the terminal latency index and residual latency, and studies have shown that they improve diagnostic accuracy in CTS (3,10,11,12). A more recent technique, median sensory latency-ulnar motor latency difference (MSUMLD), was shown to be useful in a previous small study (13). The three techniques have never been directly compared.

The aim of this study is to compare the diagnostic accuracy of all of these methods in large study involving patients with CTS of all severities.

## MATERIALS AND METHODS

### Study population

**Figure 1:**
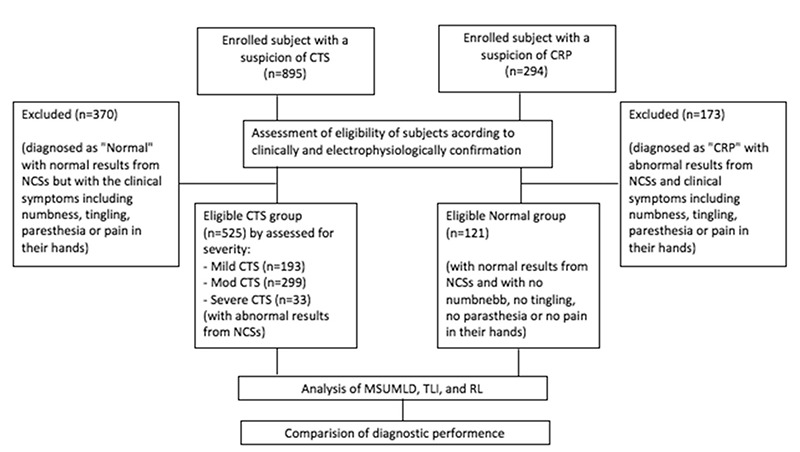
Flowchart of the study.* CRP: cervical radiculopathy; CTS: carpal tunnel syndrome; MSUMLD: median sensory-ulnar motor latency difference; NCS: nerve conduction studies; RL: residual latency; TLI: terminal latency index*

This diagnostic accuracy study was conducted retrospectively (flowchart, Figure 1). A total of 657 subjects wer enrolled between January 2012 and December 2013. The CTS group consisted of 546 subjects with clinical symptoms and findings of CTS (e.g., numbness, tingling, paresthesia, pain or sensory deficits in the median nerve distribution, weakness of the abductor pollicis brevis  muscle, and a positive Tinel’s test) and abnormal nerve conduction studies. The control group consisted of 121 subjects with clinical symptoms of cervical radiculopathy (neck pain but no hand symptoms) and normal nerve conduction studies. Patients with hand symptoms and normal nerve conduction studies were not included in either group. One hand of each subject was examined (14).

The exclusion criteria were a history of wrist fracture, previous median nerve surgery or injury, diabetes, chronic renal failure, gout, rheumatoid arthritis, thyroid disease, other systemic diseases associated with polyneuropathy, and plexopathy. The study protocol was approved by the School of Medicine’s ethics committee. Informed consent was obtained from all patients.

### Nerve conduction studies

Routine electrodiagnostic tests including sensory and motor nerve conduction studies for the median and ulnar nerves were performed using a Viasys Medelec Synergy EMG device. Skin temperature was maintained at 32.0 °C room temperature, between 22.0 and 25.0 °C. The filter bandwidth was 20 Hz-2 kHz for sensory nerve conduction studies and 10 Hz-10 kHz for motor nerve conduction studies. Sweep speed was 1 msec/division for sensory nerve conduction studies and 5 msec/division for motor nerve conduction studies. Sensitivity was 20 μV/division for both types of nerve conduction studies and increased if needed. Cup electrodes (AgCl) 8 mm in diameter were used. The distance between the recording electrodes was 3-5 cm.

The distance between stimulator electrodes was 3 cm. The stimulation intensity was 10-30 mA for sensory nerve conduction studies and 10-50 mA for motor nerve conduction studies. The duration was 0.1-0.2 msec for sensory nerve conduction studies and 0.1-0.5 msec for motor nerve conduction studies. Supramaximal stimulation was achieved by adjusting the duration and intensity of the stimulus.

Median sensory nerve conduction studies, digit II (finger)-wrist median and palm-wrist sensory nerve conduction velocities were orthodromically recorded with surface stimulation from digit II and mid palm. Latencies of the sensory nerve action potentials were measured from the onset to the initial negative peak.

The median motor compound muscle action potential (CMAP) was recorded with the active recording electrode placed over the midpoint of the abductor pollicis brevis muscle and the reference electrode placed distally over the thumb. The belly-tendon principle was followed, and the ground electrode was placed between the stimulating and recording electrodes. Median motor distal latency (mMDL) was measured from the stimulus onset to the initial CMAP response. Median motor nerve conduction velocity (mMNCV) was determined by dividing the distance between the stimulation points by the difference in conduction times.

For the ulnar motor CMAP, the active recording electrode was placed over the belly of the abductor digiti minimi (ADM) midway between the distal wrist crease and the base of digit V. The reference electrode was placed on the proximal phalanx of the digit V. Electrical stimulation of the ulnar nerve was done proximal to the active recording electrode at the wrist crease just lateral to the flexor carpi ulnaris tendon. Ulnar motor distal latency was measured from the stimulus onset to the initial ADM CMAP deflection.

Terminal latency index was calculated with Equation 1 and residual latency, with Equation 2. MSUMLD was determined by simple subtraction (13).

Terminal latency index= terminal distance / (mMNCV × mMDL)

Residual latency= mMDL - (distal distance (mm) / mMNCV)

Electrodiagnostic data were compared with normal reference values and categorized by our laboratory’s grading system (15):

- Extreme CTS (absence of motor and sensory potentials),

- Severe CTS (absence of sensory response and abnormal mMDL),

- Moderate CTS (abnormal sensory conduction combined with mMDL abnormalities),

- Mild CTS (abnormal median sensory conduction only),

- Normal (all findings in the normal range).

In extreme CTS, median sensory and motor latencies could not be obtained and hence, were not included in parameter analysis. The MSUMLD requires a median sensory response and hence, could not be determined in severe cases.

### Statistical analysis

All statistical analyses were performed using SAS v9.4 (SAS Institute, Cary, NC). Data were reported as means ± standard deviation. Means were compared using one-way ANOVA with the Bonferroni adjustment. For statistical significance, a probability level of 5% (p<0.05) was required. The sensitivity and specificity were compared using receiver operating characteristic (ROC) curve analysis.

## RESULTS

### Characteristics of the control and CTS groups

According to the standards for the reporting of diagnostic accuracy studies (16), the characteristics of the control and CTS groups are listed in Table 1. All data except gender were normally distributed. The distribution of the data was determined to be homogeneous using one-way ANOVA with the Bonferroni adjustment. The control group was significantly younger than the CTS group (p<0.05).

### Comparisons of parameters between the control and CTS groups

Comparisons of mean values between control and all CTS groups are presented in Table 2. There was a statistically significant difference in all three parameters between the control group and all CTS groups (p<0.0001).

### Comparison of sensitivity and specificity

**Figure 2:**
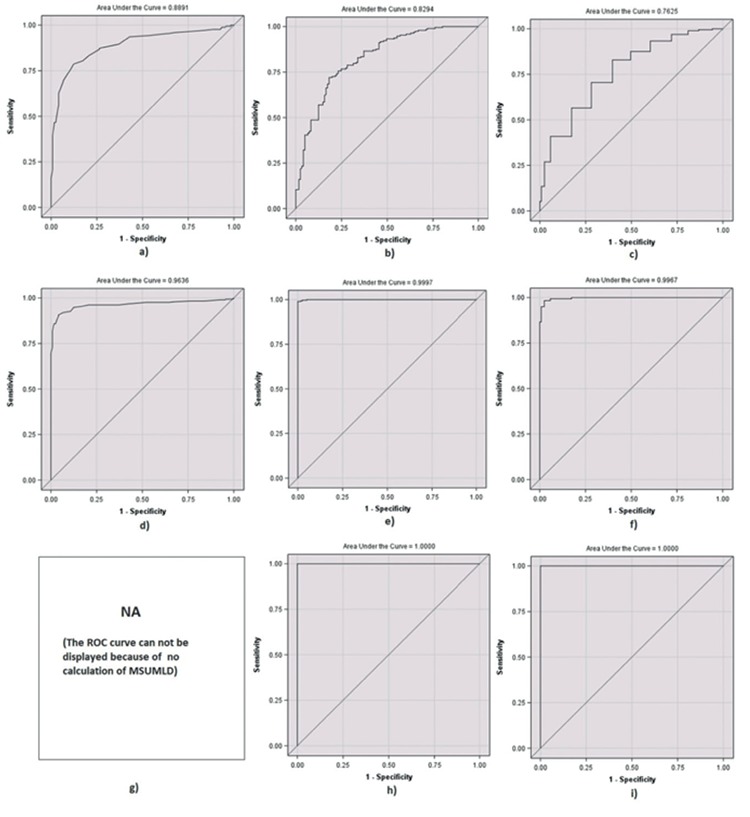
a-i. The pilot ROC curves for each parameter. Median sensory-ulnar motor latency difference for mild carpal tunnel syndrome (a); terminal latency index for mild carpal tunnel syndrome (b); residual latency for mild carpal tunnel syndrome (c); median sensory-ulnar motor latency difference for moderate carpal tunnel syndrome (d); terminal latency index for moderate carpal tunnel syndrome (e); residual latency for moderate carpal tunnel syndrome (f); median sensory-ulnar motor latency difference for severe carpal tunnel syndrome (g); terminal latency index for severe carpal tunnel syndrome (h); and residual latency for severe carpal tunnel syndrome (i).

Figure 2 shows the lowest specificity point corresponding to the highest sensitivity value of the cut-off ROC curve for each parameter. Comparing controls against all CTS patients (Table 3), using a cutoff value of >0.8 msec, the MSUMLD showed a sensitivity of 0.864, a specificity of 0.893, a positive predictive value (PPV) of 0.969, and a negative predictive value (NPV) of 0.632. Residual latency  >2.37 msec showed a sensitivity of 0.897, a specificity of 0.818, a PPV of 0.955, and a NPV of 0.647. For terminal latency index <0.26 msec, sensitivity was 0.729, specificity was 0.942, PPV was 0.983, and NPV was 0.496.

When the control and mild CTS groups were compared (Table 4), the cutoff values were slightly different. MSUMLD >1.02 msec yielded a sensitivity of 0.517, a specificity of 0.967, a PPV of 0.957, and a NPV of 0.582. For residual latency >2.4 msec, sensitivity was 0.689, specificity was 0.818, PPV was 0.858, and NPV was 0.623. For terminal latency index <0.29 msec, sensitivity was 0.829, specificity was 0.603, PPV was 0.769, and NPV was 0.689. The area under the ROC curve was the highest for the MSUMLD (0.889), followed by the residual latency (0.829), and lastly the terminal latency index (0.762), and the differences were statistically significant (MSUMLD being the most accurate).

A comparison of control and moderate CTS is shown in Table 5. For MSUMLD >0.95 msec, sensitivity was 0.866, specificity was 0.958, PPV was 0.981, NPV was 0.743, and the area beneath the ROC curve was 0.963. For residual latency >2.92, sensitivity was 0.989, specificity was 1.000, PPV was 1.000, NPV was 0.975, and the area beneath the ROC curve was 0.999. For terminal latency index <0.249, sensitivity was 0.983, specificity was 0.975, PPV was 0.989, NPV was 0.959, and the area beneath ROC curve was 0.996. Differences in the area under the ROC curve were not statistically significant, but the sensitivity of MSUMLD was lower.

Comparing controls and severe CTS (Table 6), residual latency  >3.39 yielded 1.000 for sensitivity, specificity, PPV, NPV, and area beneath the ROC curve. For terminal latency index <0.21, sensitivity was 0.969, sensitivity and PPV were both 1.000, NPV was 0.991, and area beneath the ROC curve was 1.000. Differences were not statistically significant.

## DISCUSSION

In this study, the sensitivity and specificity of terminal latency index, residual latency, and MSUMLD were examined. In CTS, conduction abnormalities are often limited to short segments of the carpal tunnel, so normal conduction in parts of the carpal tunnel can mask the slowing of conduction in mild CTS (3,12,17). This lack of sensitivity, particularly for motor conduction, may result in failure to detect abnormalities (5,18,19,20,21). Our results support earlier findings that sensory studies are of limited value in severe CTS because responses are often absent (3,22).

Previous studies showed a higher mean value of terminal latency index in the control group than our study (12,23). This may be due to the population size of the control group, gender, age distribution, and differences in the normal values that each laboratory uses. Our results showed similar terminal latency index and residual latency in CTS compared to other studies (24,25), although one showed higher sensitivity and specificity (26). This may have been due to the lack of stratification of CTS severity in some of the other studies.

The MSUMLD can be a very sensitive and specific test for CTS. It is worth noting that the MSUMLD does not require mid palm stimulation, saving time and increasing patient comfort. For MSUMLD in mild CTS, Bodofsky et al. (13) found higher sensitivity and specificity rates compared with our study. This may be due to our larger study size, as well as the generally high sensitivity rates of other techniques in more advanced cases. All the techniques worked well for moderate and severe cases, but they are usually not needed, as the diagnosis is straightforward in these cases. Mild CTS cases are difficult to diagnose, and MSUMLD is the most helpful in these cases. Ulnar motor latency is usually unaffected in mild CTS, while ulnar sensory latency rises (27,28). Previous studies have shown the median and ulnar motor latencies to be significantly correlated as well as the median and ulnar sensory latencies in both normal and CTS, while the median sensory and ulnar motor latencies are not. This can make the MSUMLD more sensitive than the (median vs ulnar) motor or sensory latency differences.

There are some limitations to this study. For severe CTS, MSUMLD could not be compared with residual latency and terminal latency index because the median sensory responses by definition could not be obtained. However, severe cases are easily diagnosed by using standard criteria. There was limited information available for some patients. This was a retrospective study. Diagnostic criteria were primarily electrodiagnostic.

There were more female than male subjects in this study. However, CTS incidence is reported to be significantly higher in the female population (29). Therefore, we did not need equal numbers of males and females in the control group to avoid bias. The younger age of the control population is another limitation, because nerve conduction velocities are affected by age. There is a negative correlation between increasing age and both NCV and amplitude per decade after the age of 20 (30). However, both median and ulnar distal latencies rise by similar degrees with increasing age, and both velocities fall to a similar degree. Therefore, a difference such as the MSUMLD should not change much with age, and this is likely also true for terminal latency index and residual latency . Our control group was referred for a clinical diagnosis of cervical radiculopathy  and was relatively younger than the CTS group. Attempting to match the CTS group by age would have required using a much smaller control group.

MSUMLD is the best calculated parameter for diagnosing mild CTS using a minimum number of tests. It requires only a simple calculation and no additional testing. Residual latency and terminal latency index are also useful in diagnosing mild to moderate CTS.

## Figures and Tables

**Table 1:**
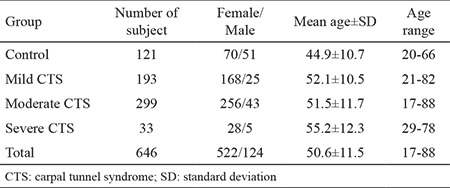
Characteristics of the control and CTS groups

**Table 2:**
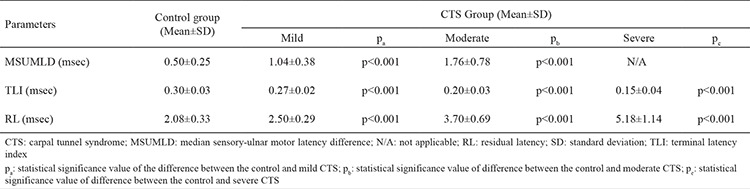
Comparison of the mean values between the control and three CTS groups

**Table 3:**
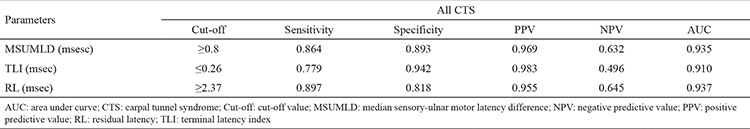
Comparison of ROC parameters between the control and all CTS group

**Table 4:**
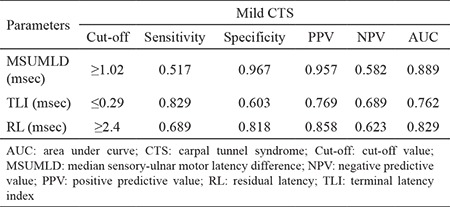
Comparison of ROC parameters between the control and mild CTS groups

**Table 5:**
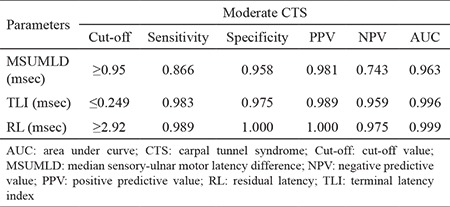
Comparison of ROC parameters between the control and moderate CTS group

**Table 6:**
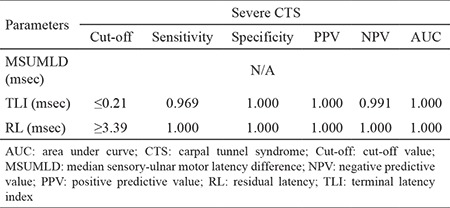
Comparison of ROC parameters between the control and severe CTS group
